# Prognostic value of neutrophil-to-lymphocyte ratio in advanced nasopharyngeal carcinoma: a large institution-based cohort study from an endemic area

**DOI:** 10.1186/s12885-018-5236-2

**Published:** 2019-01-08

**Authors:** Ji-Jin Yao, Feng-Ting Zhu, Jun Dong, Zi-Bin Liang, Le-Wei Yang, Shao-Yi Chen, Wang-Jian Zhang, Wayne R. Lawrence, Fan Zhang, Si-Yang Wang, Ying Sun, Guan-Qun Zhou

**Affiliations:** 1 Department of Radiation Oncology, Guangdong Key Laboratory of Nasopharyngeal Carcinoma Diagnosis and Therapy; Collaborative Innovation Center for Cancer Medicine, State Key Laboratory of Oncology in South China, Sun Yat-sen University Cancer Center, Guangzhou, 510060 Guangdong Province People’s Republic of China; 2 Department of Head and Neck Oncology, The Cancer Center of the Fifth Affiliated Hospital of Sun Yat-sen University, Zhuhai, 519001 Guangdong Province China; 3 Department of Imaging Diagnosis and Interventional Center, State Key Laboratory of Oncology in South China, Collaborative Innovation Center for Cancer Medicine, Sun Yat-sen University Cancer Center, Guangzhou, 510060 Guangdong Province People’s Republic of China; 4 Department of VIP Region, State Key Laboratory of Oncology in Southern China, Collaborative Innovation Center of Cancer Medicine, Sun Yat-sen University Cancer Center, Guangzhou, 510060 People’s Republic of China; 5 Department of thoracic oncology, the cancer center of the fifth affiliated hospital of Sun Yat-sen University, Zhuhai, 519001 Guangdong Province China; 6 Department of abdominal oncology, the cancer center of the fifth affiliated hospital of Sun Yat-sen University, Zhuhai, 519001 Guangdong Province China; 7 Department of Medical Statistics and Epidemiology & Health Information Research Center & Guangdong Key Laboratory of Medicine, School of Public Health, Sun Yat-sen University, Guangzhou, 510080 Guangdong Province China; 8 Department of Environmental Health Sciences, School of Public Health, University at Albany, State University of New York, New York, Rensselaer 12144 USA

**Keywords:** Nasopharyngeal carcinoma, advanced, Neutrophil-to-lymphocyte ratio, Prognosis

## Abstract

**Background:**

Findings remain unclear whether neutrophil-to-lymphocyte ratio (NLR) detrimentally affects advanced nasopharyngeal carcinoma (NPC) prognosis. We aim to evaluate the prognostic value of NLR in patients with NPC based on a large-scale cohort from an endemic area.

**Methods:**

We selected patients retrospectively from a cohort examining long-term cancer outcomes following diagnosis. Neutrophil counts and lymphocyte counts were assessed prior to treatment. Kaplan–Meier method and log-rank test were used to calculate and compare survival outcomes. Additionally, Cox proportional hazards model was utilized to carry out univariate and multivariate analyses.

**Results:**

Between October 2009 and August 2012, we enrolled 1550 consecutive NPC patients staged II-IVB. The median value of NLR was 2.27 (interquartile range [IQR], 1.71–3.12). Determined by operating characteristic curve using overall survival (OS) as an endpoint, the cutoff value for NLR was 2.50. At 5 years, NLR > 2.50 was associated with inferior OS (90.3% vs 82.5%; *P* < 0.001), distant metastasis-free survival (DMFS, 89.4% vs 85.0%; *P* = 0.014), and progression-free survival (PFS, 80.9% vs 76.5%; *P* = 0.031) than NLR ≤2.50. In multivariate analysis, NLR was found to be a significant prognostic factor for OS (HR, 1.72; 95% CI, 131–2.24; *P* < 0.001), DMFS (HR, 1.45; 95% CI, 1.10–1.92; *P* = 0.009), and PFS (HR, 1.29; 95% CI, 1.04–1.59; *P* = 0.021).

**Conclusion:**

Pretreatment NLR independently affects survival. Our findings suggest that NLR measurements will be of great clinical significance in the management of NPC.

## Background

In Southern China, nasopharyngeal carcinoma (NPC) is a common malignancy. Previous literature reported the annual incidence rate varied from 15 to 50 cases per 100,000 [1]. Tumor-Node-Metastasis (TNM) stage is currently the primary measure to predict NPC prognosis. However, TNM staging system is not adequate for predicting NPC outcome, and patients that are in the same TNM stage often have substantial clinical heterogeneity [2]. Plasma epstein–barr virus (EBV) DNA titre remains the sole biomarker that has clinical utility in patients with NPC [3, 4]. Nevertheless, the high cost and great inter-laboratory variability for examination of plasma EBV DNA hinders the ability to include in routine clinical practice [5]. For this reason, it is important to identify inexpensive, objective, and easily detected markers to complement NPC prognosis TNM classification system.

Prior literature has shown systemic inflammatory response stimulates cancer metastasis and progression by facilitating angiogenesis and inhibiting apoptosis [6]. An important biomarker is the neutrophil to lymphocyte ratio (NLR), which could accurately show systemic inflammation [7]. The association between elevated NLR on adverse prognoses was reported for multiple tumor types [8]. In clinical settings, neutrophil and lymphocyte counts in peripheral blood are routinely measured, where for the calculation of NLR, additional effort are needed. Thus, NLR is potentially a promising prognostic biomarker for NPC. To date, several studies have examined the prognostic value of NLR in NPC patients [9–13]. However, the results of these studies have been inconsistent, and the prognostic role of NLR for NPC have not been conclusively determined.

To fill this gap in knowledge, we investigated the long-term prognostic effect of NLR on the outcome of patients with NPC using a large-scale homogenous patient cohort.

## Methods

### Patient population

NPC patients treated by radiotherapy with curative intent from October 2009 through August 2012 were identified. This retrospective study with prospectively collected data included a cohort of 1550 men and women. Patients were included if (1) histologically confirmed NPC; (2) had no prior history of malignancy; (3) absence of distant metastasis; (4) stage II-IVA disease according to the 8th edition of the American Joint Committee on Cancer (AJCC) staging system; (5) did not receive prior treatment for NPC; (6) complete pretreatment history of hematological variables; and (7) no infection or inflammatory conditions.

### Pretreatment evaluation

All included patents had undergone routine pretreatment evaluations comprising of medical history, complete physical examination, complete blood count, fiber-optic nasopharyngoscopy, chest radiography, abdominal sonography, magnetic resonance imaging (MRI) of the nasopharynx and neck, and bone scan or whole-body fluorodeoxyglucose positron emission tomography. Patients were restaged in accordance to the 8th edition of the AJCC staging system for NPC [14]. The present study was conducted in adherence with institutional policies to protect confidential material including all patients’ information, and approved by our Institutional Review Board. We uploaded the key raw data onto the Research Data Deposit (RDD) public platform (http://www.researchdata.org.cn), and was assigned the RDD approval number RDDA2017000386. If someone need to access the data, he/she should obtain our consent, and have to explain the source of the data in their study.

### Laboratory examination

Absolute neutrophil and lymphocyte counts were assessed before treatment and determined utilizing a Sysmex XE-5000 automated hematology analyzer (Sysmex, Kobe, Japan). We calculated NLR as the absolute counts of neutrophil divided by the absolute lymphocyte counts.

### Treatment

Intensity-modulated radiotherapy (IMRT) was used for treating primary tumor and the upper neck area above the caudal edge of the cricoid cartilage. Using a previously described treatment protocol by our institution, target volumes were delineated [15] in agreement with the International Commission on Radiation Units (ICRU) and Measurements reports 62 [16] and 83 [17]. Our institutional guidelines during the study period was in accordance to the 7th edition of the AJCC staging system which suggested concurrent chemoradiotherapy (CCRT) for stage II disease, and CCRT +/− neoadjuvant and adjuvant chemotherapy for stages III to IVB NPC. Neoadjuvant or adjuvant chemotherapy contained 5-fluorouracil (800 mg/m^2^/day over 120 h) with cisplatin (80 mg/m^2^), or cisplatin (80 mg/m^2^) with docetaxel (80 mg/m^2^) administered at three week intervals for 3 cycles. Concurrent chemotherapy consisted of cisplatin (80 or 100 mg/m^2^) given in weeks 1, 4, and 7 of RT, or cisplatin (40 mg/m^2^) given weekly during RT, beginning on the first day of RT.

### Outcome and follow-up

We selected the primary endpoint as overall survival (OS), and secondary endpoints included distant metastasis-free survival (DMFS), locoregional relapse-free survival (LRFS), and progression-free survival (PFS). We calculated overall survival from initial treatment to death. For distant and locoregional relapse-free survival analyses, we recorded the latencies (i.e. time from initial treatment) to the first remote or locoregional relapse respectively. We calculated progression-free survival from the date of initial treatment to the date of treatment failure or death from any cause, whichever was first. Patients were seen every three months during the first 2 years, every six months for years 3 through 5, and annually thereafter until death. The duration of patient follow-up was measured from the first day of therapy to either the day of last examination or the day of death.

### Statistical analysis

For categorical variables, we calculated relative frequencies (percentage), while for continuous variables we calculated median (interquartile range [IQR]). Additionally, categorical variables were compared using χ^2^ test. Receiver operating characteristic (ROC) curve analysis was used to evaluate the cutoff point for NLR. Cumulative survival rates were depicted by Kaplan–Meier curves and compared by Log-rank tests for each dichotomized biomarker. Univariate and multivariate analysis utilizing a Cox proportional hazards model was used to test the independent association of different factors by backward elimination. All statistical analysis were 2-tailed, and *P* < 0.05 was determined statistically significant. All statistical analyses were completed using R 3.1.2.

## Results

### Patient characteristics

Table 1 presents the characteristics of 1550 patients with NPC that met the inclusion criteria. The median age was 45 years (range, 14–78 years). During the median follow-up duration of 54.3 months (IQR, 1.3–85.6 months), 224 patients died, including 153 due to distant metastases, 45 because of local and/or regional relapse, 11 as a result of non-cancer causes, 6 from secondary malignant tumors, and 9 due to unknown causes. Additionally, 200 patients developed distant metastases, and 104 experienced local or regional relapse. The 5-year survival rates among patients were OS, 85.3%; DMFS, 87.7%; LRFS, 90.1%; and PFS, 78.3%.

**Table 1:** Characteristics of 1550 patients

Characteristic	No. of patients (%)
Age, yr	
Median	45
Interquartile range	38–53
Sex	
Male	1167 (75.3)
Female	383 (24.7)
T stage	
T1	190 (12.3)
T2	265 (17.1)
T3	796 (51.4)
T4	299 (19.3)
N stage	
N0	190 (12.3)
N1	928 (59.9)
N2	226 (14.6)
N3	206 (13.3)
Overall stage	
II	332 (21.4)
III	754 (48.6)
IVA-B	464 (29.9)
Family history	
No	444 (28.6)
Yes	1106 (71.4)
Smoking history	
No	961 (62.0)
Yes	589 (38.0)
Drinking history	
No	1358 (87.6)
Yes	192 (12.4)
NLR	
Median	2.27
Interquartile range	1.71–3.12
Chemotherapy	
RT alone	165 (10.6)
CCRT	594 (38.3)
NACT+CCRT	756 (48.8)
CCRT+AC	35 (2.3)

Abbreviation: *RT* radiotherapy, *CCRT* concurrent chemoradiotherapy, *NACT* neoadjuvant chemotherapy, *AC* adjuvant chemotherapy, *NLR* neutrophil-to-lymphocyte ratio

### The prognostic value of NLR in NPC

**Fig. 1:**
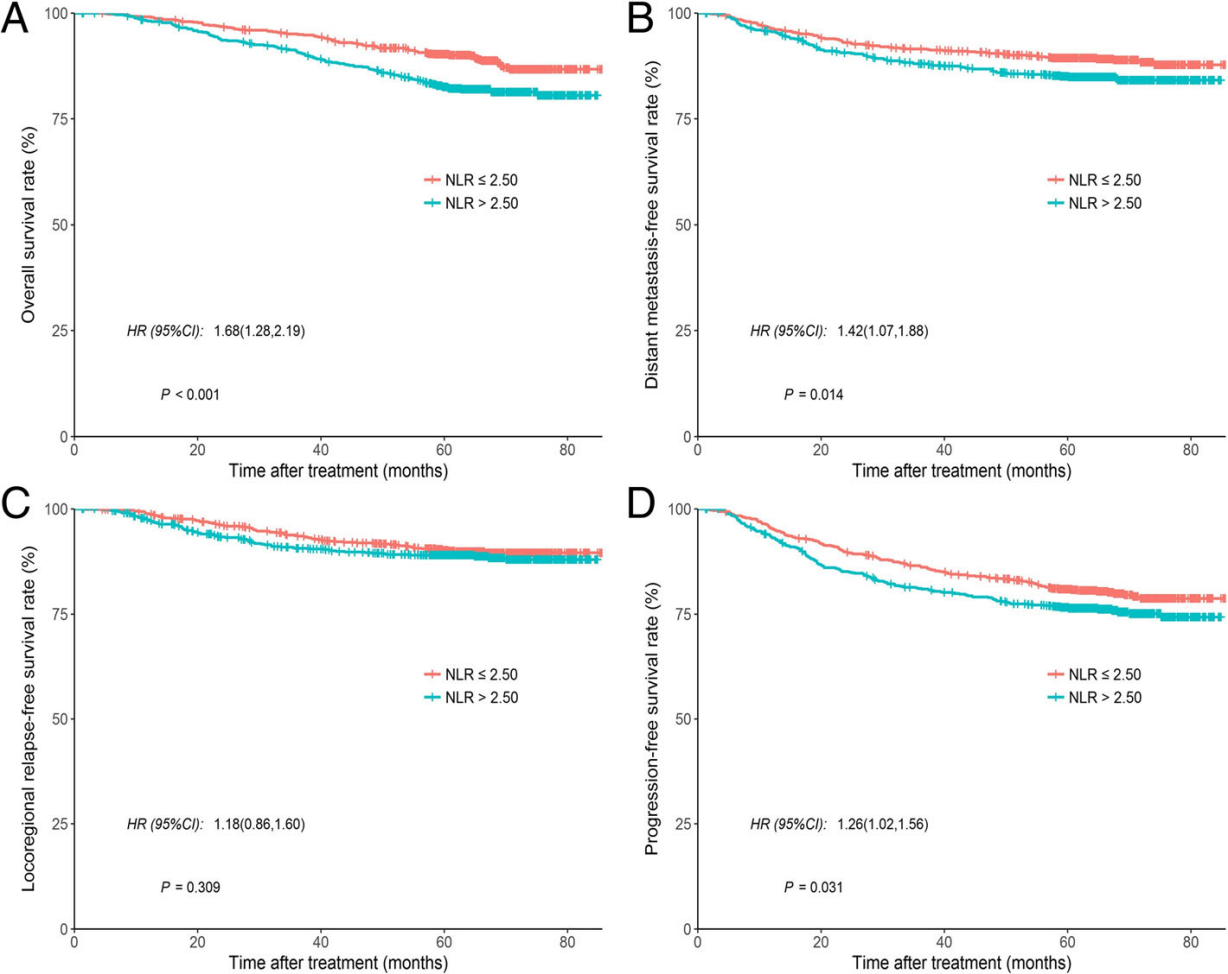
According to NLR determined by ROC (≤ 2.50 vs. > 2.50), Kaplan-Meier survival curves for (**a**) overall survival, (**b**) distant metastasis-free survival, (**c**) locoregional recurrence-free survival, and (**d**) progression-free survival

The median value of NLR was 2.27 (IQR, 1.71–3.12). Using OS as the endpoint, the cutoff value for NLR was 2.50, determined using ROC curve. At 5 years, patients with NLR >  2.50 had significantly inferior OS (90.3% vs 82.5%; *P* < 0.001) (Fig. 1a), DMFS (89.4% vs 85.0%; *P* = 0.014) (Fig. 1b), and PFS (80.9% vs 76.5%; *P* = 0.031) (Fig. 1d) than patients with NLR ≤ 2.50. Nevertheless, we did not observe any difference in LRFS between patients with NLR ≤ 2.50 and NLR >  2.50 (90.2% vs. 89.0%, *P* = 0.309; Fig. 1c). Further multivariate analysis revealed that age, T stage, N stage, and NLR were associated with both OS and PFS (*P* < 0.05 for all). N stage (HR, 3.50; 95% CI, 2.65–4.63; *P* < 0.001) and NLR (HR, 1.45; 95% CI, 1.10–1.92; *P* = 0.009) were also associated with DMFS; and only age (HR, 1.40; 95% CI, 1.02–1.92; *P* = 0.035) was associated with LRFS (Table 2). In order to further demonstrate the predictive value of NLR in advanced NPC, we also analyzed the prognostic factors for advanced NPC in the multivariate models without NLR. Our results showed that age, T stage, and N stage were independent risk factors for both OS and PFS (*P* < 0.05 for all). Additionally, N stage (HR, 3.49; 95% CI, 2.64–4.61; *P* < 0.001) was an independent risk factor for DMFS; and age was a significant predictor for LRFS (HR, 1.40; 95% CI, 1.02–1.92; *P* = 0.035) (Table 3). Overall, the C-index of multivariate model with or without NLR were 0.69 (95% CI, 0.65–0.73) and 0.68 (95% CI, 0.64–0.72), 0.67 (95% CI, 0.64–0.71) and 0.67 (95% CI, 0.63–0.70), 0.57 (95% CI, 0.52–0.61) and 0.55 (95% CI, 0.51–0.60), and 0.63 (95% CI, 0.60–0.66) and 0.63 (95% CI, 0.60–0.66) for OS’s, DMFS’s, LRFS’s, and PFS’s multivariate model, respectively.

**Table 2:** Multivariate analysis of NLR determined by ROC for patients with advanced NPC

Endpoint	Variable	HR	95% CI for HR	*P* value
OS				
	Age (≤ 45 vs. > 45)	1.69	1.29–2.22	< 0.001
	Sex (Male vs. Female)	1.30	0.90–1.87	0.163
	Smoking history (No vs. Yes)	1.11	0.83–1.49	0.492
	T stage (T1–2 vs. T3–4)	1.34	0.98–1.85	0.071
	N stage (N0–1 vs. N2–3)	2.86	2.19–3.73	< 0.001
	NLR (≤ 2.50 vs. > 2.50)	1.72	1.31–2.24	< 0.001
	Treatment (RT alone vs CCRT)	1.43	0.82–2.50	0.213
	Treatment (RT alone vs NACT+CCRT)	1.23	0.70–2.14	0.472
DMFS				
	Sex (Male vs. Female)	1.34	0.95–1.90	0.097
	N stage (N0–1 vs. N2–3)	3.50	2.65–4.63	< 0.001
	NLR (≤ 2.50 vs. > 2.50)	1.45	1.10–1.92	0.009
LRFS				
	Age (≤ 45 vs. > 45)	1.40	1.02–1.92	0.035
	N stage (N0–1 vs. N2–3)	1.35	0.97–1.88	0.077
PFS				
	Age (≤ 45 vs. > 45)	1.44	1.16–1.79	0.001
	Family history (No vs. Yes)	1.17	0.91–1.50	0.213
	Smoking history (No vs. Yes)	1.12	0.90–1.39	0.323
	T stage (T1–2 vs. T3–4)	1.27	0.99–1.62	0.063
	N stage (N0–1 vs. N2–3)	2.25	1.81–2.79	< 0.001
	NLR (≤ 2.50 vs. > 2.50)	1.29	1.04–1.59	0.021

Abbreviation: *OS* overall survival, *DMFS* distant metastasis-free survival, *LRFS* locoregional relapse-free survival, *PFS* progression-free survival, *RT* radiotherapy, *CCRT* concurrent chemoradiotherapy, *NACT* neoadjuvant chemotherapy, *NLR* neutrophil-to-lymphocyte ratio, *HR* rate ratio, *CI* confidence interval

**Table 3:** Multivariate models without NLR for patients with advanced NPC

Endpoint	Variable	HR	95% CI for HR	*P* value
OS				
	Age (≤ 45 vs. > 45)	1.64	1.25–2.14	< 0.001
	Sex (Male vs. Female)	1.3	0.90–1.88	0.158
	Smoking history (No vs. Yes)	1.09	0.82–1.46	0.553
	T stage (T1–2 vs. T3–4)	1.42	1.04–1.94	0.029
	N stage (N0–1 vs. N2–3)	2.84	2.18–3.69	< 0.001
DMFS				
	Sex (Male vs. Female)	1.34	0.95–1.89	0.098
	N stage (N0–1 vs. N2–3)	3.49	2.64–4.61	< 0.001
LRFS				
	Age (≤ 45 vs. > 45)	1.4	1.02–1.92	0.035
	N stage (N0–1 vs. N2–3)	1.35	0.97–1.88	0.077
PFS				
	Age (≤ 45 vs. > 45)	1.42	1.15–1.77	0.001
	Family history (No vs. Yes)	1.17	0.92–1.50	0.205
	Smoking history (No vs. Yes)	1.11	0.89–1.38	0.34
	T stage (T1–2 vs. T3–4)	1.28	1.00–1.65	0.048
	N stage (N0–1 vs. N2–3)	2.24	1.81–2.78	< 0.001

Abbreviation: *OS* overall survival, *DMFS* distant metastasis-free survival, *LRFS* locoregional relapse-free survival, *PFS* progression-free survival, *RT* radiotherapy, *CCRT* concurrent chemoradiotherapy, *NACT* neoadjuvant chemotherapy, *NLR* neutrophil-to-lymphocyte ratio, *HR* rate ratio, *CI* confidence interval

### NLR is associated with known prognostic clinical indices

In the present study, ROC curve was used to evaluate different cutoff points for NLR. As previously described, we divided patients into two groups according to NLR: high NLR (> 2.50) and low NLR (≤ 2.50). Additionally, we examined the correlations between NLR and various clinicopathological features. Patients that smoked generally had higher NLR (*P* = 0.011) and also patients with advanced disease (higher T stage, *P* = 0.001; N stage, *P* = 0.015; overall stage, *P* < 0.001). However, no significant differences were observed between groups regarding age, sex, family history, history of alcohol consumption, or treatment strategy (all *P* > 0.05; Table 4).

**Table 4:** Baseline characteristics of the patients with advanced NPC stratified by NLR

	No. of patients (%) stratified by NLR		
Characteristic	≤ 2.50 (*n* = 813)	> 2.50 (*n* = 737)	*P* value
Age			0.222
≤ 45	404 (49.7)	343 (46.6)	
> 45	409 (50.3)	394 (53.4)	
Sex			0.034
Male	575 (70.7)	557 (75.6)	
Female	238 (29.3)	180 (24.4)	
T stage			0.001
T1	110 (13.5)	80 (10.9)	
T2	151 (18.6)	114 (15.5)	
T3	418 (51.4)	378 (51.3)	
T4	134 (16.5)	165 (22.4)	
N stage			0.015
N0	106 (13.0)	84 (11.4)	
N1	485 (59.7)	443 (60.1)	
N2	132 (16.2)	94 (12.8)	
N3	90 (11.1)	116 (15.7)	
Overall stage			< 0.001
II	189 (10.9)	143 (19.4)	
III	316 (38.9)	341 (46.3)	
IVA-B	396 (48.7)	253 (34.3)	
Family history			0.574
No	575 (70.7)	531 (72.0)	
Yes	238 (29.3)	206 (28.0)	
Smoking history			0.402
No	496 (61.0)	465 (63.1)	
Yes	317 (39.0)	272 (36.9)	
Drinking history			0.247
No	720 (88.6)	638 (86.6)	
Yes	93 (11.4)	99 (13.4)	
Treatment strategy			0.779
RT	89 (10.9)	76 (10.3)	
CCRT alone	316 (38.9)	278 (37.7)	
NACT+CCRT	408 (50.2)	383 (52.0)	

Abbreviation: *NLR* neutrophil-to-lymphocyte ratio, *CCRT* concurrent chemoradiotherapy, *NACT* neoadjuvant chemotherapy, *RT* radiotherapy

## Discussion

Although an elevated ratio of NLR was reported as an inadequate prognostic indicator in numerous cancers [18], the prognostic ability of NLR is not conclusively determined for NPC. Our analysis from a large sample indicated that patients with NLR > 2.50 were generally associated with inferior OS, DMFS, and PFS, compared to patients with NLR ≤2.50, except for LRFS. Further analyses to detect interactions between NLR and clinicopathological characteristics found that among patients that either smoked or had further advanced disease (higher T stage, N stage, and overall stage) were also more likely to have high levels of NLR.

In an analysis by Templeton and colleagues [19] on NLR as a prognostic biomarker, suggested that high NLR was associated with adverse survival regardless of the threshold for patient stratification. Recently, An et al. [9] retrospectively reviewed 363 NPC patients, and suggested that a high NLR > 3.73 was strongly associated with inferior PFS, DMFS, and LRFS for NPC patients. Another study reported by Sun et al. [10] indicated that NLR ≥2.7 was associated with shorter PFS in patients with NPC. In the present study, multivariate analysis showed that increasing NLR > 2.50 was mostly detrimental to OS, DMFS, and PFS. Consistent with our study, Li et al. [18] prospectively analyzed the prognostic value of inflammatory biomarkers in a cohort of NPC patients (*N* = 388), and indicated that NLR > 2.50 was significantly associated with inferior PFS.

Conversely, Chua et al. [11] examined the significance of NLR prognostic in a pooled cohort of NPC patients (*N* = 380) from two controlled trials, but were unable to determine if NLR adds prognostic value for NPC. The first potential reasoning is that different NLR levels have different prognostic value. In the study by Chua et al., NLR was dichotomized into binary variables using the median value of NLR stratified patients, but failed to use ROC curve, which is confirmed to be a central or unifying position in the process of assessing and using diagnostic tool to analyze the optimum cutoff point [20, 21]. Secondly, the study was underpowered because of the long duration of 15 years to recruit patients, potentially leading to inter-study heterogeneity, specifically data maturity and quality of radiotherapy techniques as highlighted by the authors. Third, although Chua et al. [11] found patients with high NLR tended to have lower survival, this trend did not reach statistical significance. This could be due to the inability to identify an effect because of small sample size.

Elevated NLR was recognized as a significant risk factor in patients with NPC. However, the mechanisms underlying this observation remains largely unclear. One possibility might be that high NLR serves as a marker for up-regulated inflammatory processes within the host microenvironment that potentially promote the development of more aggressive tumor clones [22–24]. Another potential reason is that elevated markers for systemic inflammatory response may increase with elevated circulating concentrations of several cytokines (IL-6, IL-7, IL-8, IL-9, IL-12, IL-1ra). Of these cytokines, IL-6 in particular acts to increase the synthesis of acute-phase proteins, and have shown to be associated with both adverse prognosis and tumor stage in several types of cancers [25].

The associations between NLR and TNM stage have previously been reported. Based on findings from prior studies that reported a positive association of NLR with TNM stage [11], we applied a threshold value of NLR > 2.50 for stratifying patients. This stratification allowed us to identify that patients with NLR > 2.50 were strongly associated with more advanced disease (higher T stage, *P* = 0.001; N stage, *P* = 0.015; overall stage, *P* < 0.001). This suggests NLR contributes to patient stratification by providing additional information about disease burden. Of interest, our results indicate that individuals that smoke commonly had higher levels of NLR. It is plausible that the variations of NLR were influenced by smoking-related inflammation [26].

A major limitation in the present study is that we did not collect information on other hematologic markers of inflammation, such as lymphocyte-monocyte ratio (LMR) and C-reactive protein [27, 28]. Another limitation is that although several threshold values were used and validated as the cutoff for NLR, not all cutoff values were proven significant. Moreover, different research institutions use varied levels of NLR, including 2.50 [18, 29], 2.75 [10], and 3.00 [11]. This inconsistency might be due to obvious heterogeneity between patients within these studies. However, we must note that NLR varied significantly for T-stage and N-stage, and overall tumor classification stage [11].

## Conclusions

In summary, pretreatment NLR independently affects survival for advanced NPC. Increasing NLR > 2.50 was mostly detrimental to OS, DMFS, and PFS in patients with advanced NPC. Additionally, pretreatment NLR may serve as a cost-effective prognostic factor in patients with NPC, and pretreatment NLR measurements will be of great clinical significance in the management of NPC.

## Abbreviations

AJCC: American Joint Committee on Cancer
CCRT: Concurrent chemoradiotherapy
DMFS: Distant metastasis-free survival
EBV: Epstein–barr virus
ICRU: International Commission on Radiation Units
IMRT: Intensity-modulated radiotherapy
IQR: Interquartile range
LRFS: Locoregional relapse-free survival
NLR: Neutrophil-to-lymphocyte ratio
NPC: Nasopharyngeal carcinoma
OS: overall survival
PFS: Progression-free survival
RDD: Research Data Deposit
ROC: Receiver operating characteristic
TNM: Tumor-Node-Metastasis
